# Methods for measuring neural activity during voluntary wheel running

**DOI:** 10.1016/j.jneumeth.2026.110839

**Published:** 2026-06-25

**Authors:** Ayland C. Letsinger, Bryan N. Ochoa, Jessica J. Wu, Kayen U. Tang, Diane Youngstrom, Shaohua Wang, Matt Bridge, Guohong Cui, Jerrel L. Yakel

**Affiliations:** a Neurobiology Laboratory, National Institute of Environmental Health Sciences, National Institutes of Health, Durham, NC, USA; b The Department of Kinesiology and Health Education, University of Texas at Austin, Austin, TX, USA; c DLH, LLC, Bethesda, MD, USA

**Keywords:** Fiber photometry, Voluntary wheel running, Cholinergic, DeepLabCut, SimBA, Ventral dentate gyrus, Physical activity neurobiology

## Abstract

**Background:**

Rodent wheel running provides a translational model to study the neurobiology of physical activity, including motivation, affect, and plasticity. However, the voluntary and unconstrained nature of wheel running makes precise behavior-to-signal alignment technically challenging.

**New method:**

We present a workflow that aligns fiber photometry signals with pose-derived behavior during voluntary wheel running. As a use case, we record acetylcholine activity in the ventral dentate gyrus of mature male C57BL/6 J mice and integrate pose estimation (DeepLabCut), supervised behavior classification (SimBA), spectral/event processing (FiPhA), and custom within-event trend estimations (R).

**Results:**

In this proof-of-concept application, acetylcholine in the ventral dentate gyrus appeared to increase 0–5 s before and throughout wheel running events during both acquisition and maintenance phases. Acetylcholine levels also showed a positive correlation with off-wheel body length.

**Comparison with existing methods:**

Prior studies measuring neural activity during physical activity have relied on head-fixation or forced treadmill running, which introduce stress confounds and reduce ecological validity, or wheel rotational velocity signals, which cannot distinguish active running from passive wheel rotation without manual annotation of video frames. The present workflow addresses these limitations by using voluntary homecage wheel running to minimize stress and supervised machine learning classification that reduces behavioral annotation time by an estimated 90% while achieving greater than 96% precision and recall.

**Conclusions:**

This workflow provides a template for efficiently and accurately aligning wheel running behavior with neural *in vivo* signals. Our proof-of-concept demonstrates the feasibility of generalizing the approach to other neuromodulators, brain regions, and recording modalities.

## 1. Introduction

Rodent voluntary wheel running can be used as a translational assay for studying the biological mechanisms of physical activity behavior including motivation, affect, memory, and cognition (Greenwood and Fleshner, 2019; Manzanares et al., 2019; Richter et al., 2014; Sherwin, 1998). Functionally, the assay is best utilized to gain insights in exercise addiction as inbred mice run an average 210.1 min (SD ± 125.2 min) in a 24-hour period (Lightfoot et al., 2010) roughly seven times the recommended guidelines for humans. This extreme level of physical activity is likely reinforced by an intrinsic positive affect, a hypothesis supported by evidence of wheel running-related anxiolysis, place preference for running wheels, withdrawal-like stereotypic behavior (*e*.*g*., chewing or excessive locomotion) when running wheels are removed or locked, preference for running wheels over drugs of abuse or high fat foods, neural activity akin to addiction, and the potential to run to death in models of anorexia nervosa (Greenwood et al., 2011; Kanarek et al., 2009; Sciolino and Holmes, 2012; Tesmer et al., 2024). Given that most humans struggle to sustain consistent, long-term exercise behavior (Troiano et al., 2008), even when intentions are present (Bauman et al., 2012; Sheeran and Webb, 2016), elucidating the neurobiological mechanisms underlying voluntary wheel running in rodents will offer valuable translational insights.

The trajectory of physical activity behaviors is complex but can be delineated into two distinct phases: acquisition and maintenance. The acquisition phase is characterized by the initial exposure to the activity, during which the organism learns the motor skills and contingencies necessary for its consistent and reliable execution. In the context of voluntary wheel running in rodents, acquisition typically manifests as a progressive increase in daily running distance, duration, and speed over a period ranging from 5 to 21 days, contingent upon the specific experimental paradigm and rodent strain. Subsequently, the maintenance phase ensues defined by a consistent daily wheel running phenotype (e.g., speed, duration, and distance). In contrast to rodent models, the maintenance of physical activity in most humans is particularly vulnerable to attrition (Rhodes and Sui, 2021). Rodents typically demonstrate a sustained high level of wheel running throughout their lifespan, exhibiting only gradual declines rather than complete cessation of the behavior over time. The neurotransmission present in both phases is essential for understanding the processes by which physical activity behaviors are reinforced. The hippocampus is one brain region that has consistently been found to be responsive to physical activity (Rhodes et al., 2003). Modulating hippocampal GABAergic or glutamatergic innervation alters movement velocity in rodents (Bender et al., 2015; Fuhrmann et al., 2015). Hippocampal acetylcholine activity, released from basal forebrain projections, stimulates plasticity throughout the hippocampus (Letsinger et al., 2021). However, it is not known how these signals change throughout acquisition and maintenance.

The neural correlates of physical activity, including acetylcholine within the hippocampus, have been studied using *in vivo* miniature microscopes (Dombeck et al., 2007; Ozbay et al., 2018; Xuan et al., 2025a), fiber photometry (Azcorra, 2023; Moghadam et al., 2025; Nishitani et al., 2025; van der Zouwen et al., 2025; Walle et al., 2024), microdialysis (Meeusen et al., 2001; Zhang et al., 2017), local field potentials (Fisher et al., 2016; Liu et al., 2024), fast-scan voltammetry (Greenwood et al., 2011), and optogenetics (Cassity et al., 2024). These studies have added tremendous understanding into the neurobiological mechanisms related to movement and motivation. However, all studies to date have utilized either head fixed mice, forced locomotion on treadmills, or voluntary locomotion movement in closed running wheels or operant chambers - all of which fall short of capturing ecological voluntary physical activity while adding confounding levels of anxiety (Dishman, 1997; Svensson et al., 2016). While more ecologically valid (Meijer and Robbers, 2014), measuring neural activity during voluntary wheel running presents a precise signal-to-behavior alignment challenge as rotational velocity of a running wheel cannot distinguish active locomotion from passive rotation due to inertia when a mouse exits the wheel (*i.e*., the mouse may terminate running while the wheel still registers movement). Overcoming this accuracy challenge requires time consuming frame by frame annotation. Therefore, we sought to create a methodological workflow for generating high fidelity recordings of neurotransmission during true voluntary wheel running (*i.e*., uninstructed, self-initiated locomotion on a running wheel within the home cage) using supervised machine learning allowing high precision in considerably less time. In this manuscript, we present sub-second recordings of acetylcholine levels within the ventral dentate gyrus during two phases of essential wheel running behavior - acquisition and maintenance, using our novel combination of *in vivo* techniques and machine learning.

## 2. Methods

### 2.1. Animals

**Fig. 1.**
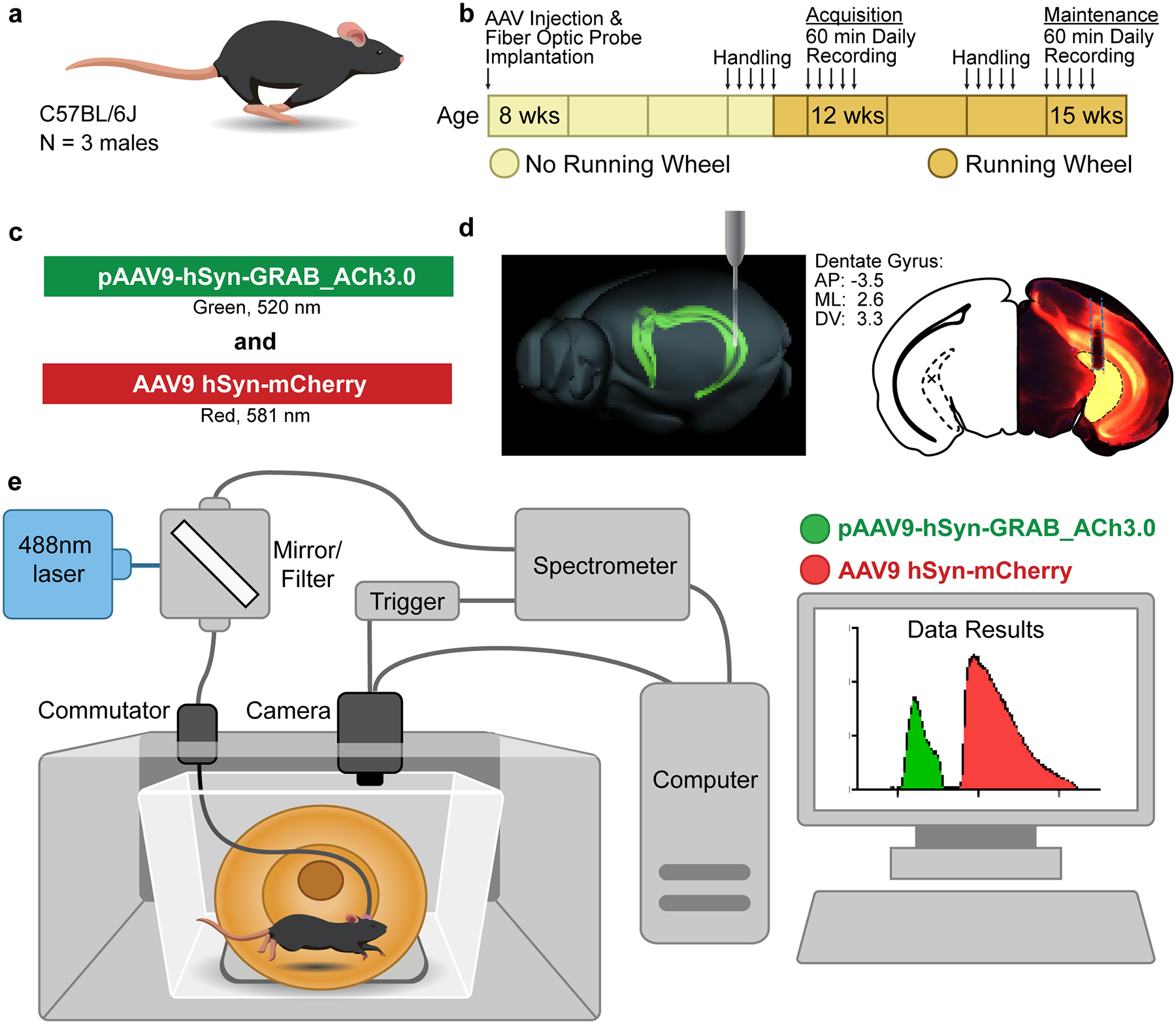
Fiber photometry workflow enables synchronized acetylcholine activity recording during voluntary wheel running. a) C57BL/6 J mice. N = 3 males for final trend analyses; N = 4 males and 4 females for behavior-classification training. b) AAV injection and fiber implantation at 8 weeks of age. 60 min daily recording at 12 weeks of age for acquisition and 15 weeks of age for maintenance. Darker yellow rectangles indicate free running wheel access. c) pAAV9-hSyn-GRAB_ACh3.0_ (green; peak emission ~520 nm) and AAV9-hSyn-mCherry (red; peak emission ~581 nm). d) On the left is an illustration of the optimal optic fiber implantation above the ventral dentate gyrus (coordinates from bregma: AP −3.5, ML 2.6, DV 3.3 mm). On the right is a representative expression image shown with spread of mCherry apparent throughout hippocampus. e) 488 nm excitation delivered through mirrors/filters to the implanted fiber via commutator; emitted fluorescence returned to a spectrometer synchronized with the behavior camera by a TTL trigger (25 Hz). Example green/red emission traces are shown.

All procedures were approved and performed in compliance with the NIEHS/ NIH Humane Care and Use of Animals Protocols (2019-0043).. Male and female C57BL/6 J mice (N = 8) were purchased at 8 weeks of age (The Jackson Laboratory) and were singly housed in rat cages maintained on a reverse 12-hour light/12-hour dark cycle with lights off at 9 a.m. (Fig. 1a). Cages were enriched with an orange dome, white cotton pads, and brown paper strips. Food and water were provided *ad libitum*.

### 2.2. Virus injection and optical fiber implants

At 8 weeks of age, mice were weighed and anesthetized using an intraperitoneal injection of ketamine/xylazine (10 mg/ml Ketamine & 0.7 mg/ml Xylazine) at a dosage of 0.1 ml per 10 g of body mass (Fig. 1b). Once unresponsive to toe pinches, the scalp was shaved, and the mouse was placed in a stereotaxic frame with continuous application of 0.25–1.25% isoflurane at a flow rate of 0.75 l/min, adjusted as needed based on regular 5-minute breath and toe pinch reflex checks. Mice were given 1 ml of saline subcutaneously in the flanks for hydration. The scalp was cleaned using iodine swabs, and 0.01 ml of 5 mg/ml Bupivacaine was injected subcutaneously beneath the scalp to provide local analgesia before an incision was performed. Cranial sutures were revealed using a sterile cotton swab lightly damp with hydrogen peroxide. After correcting for tilt and scaling using bregma and lambda, cranial holes were drilled with 1-gauge drill bits. For viral injections (Fig. 1c), a volume of 500 nl was delivered at a rate of 100 nl/min using a calibrated syringe (10 μL, Microliter Syringe, Cemented Needle, PN: 80300; Hamilton Company) in the ventral dentate gyrus (Fig. 1d; from bregma AP: −3.5, ML: 2.6, DV: 3.3). The viral cocktail was a 10:1 solution of 1.02E13 GC/ml pAAV9-hSyn-GRAB_ACh3.0_ and 6.00E12 GC/ml pAAV-hSyn-mCherry (Addgene, packaged by the NIEHS Viral Vector Core). The syringe was left in place for 3 min after injection to minimize backflow of virus upon removal. A fiber optic cannula (5 mm length fiber optic, 200 μm diameter fiber optic core, 0.38 numerical aperture, 6.4 mm length cannula, 1.25 mm diameter cannula; Thor Inc.) was fixed 500 μm above the target region using dental cement (C&B-Metabond, Patterson Dental Company). To reduce postoperative pain, 0.02 ml per 10 g body mass of Buprenorphine (0.5 mg/ml) was injected subcutaneously in the flank. Mice were given 0.1 ml of intraperitoneal Atipamezole (0.16 mg/ml) as an antisedative. Mice were returned to their home cage on a heating pad until ambulatory and were given one month to recover and allow maximal viral expression before entering experimental timelines. Mice have been found to fully recover (*i*.*e*., return to previous levels of wheel running) from intracranial injections within one week (Supplemental Figure 1).

### 2.3. Confirmation of viral expression and fiber optic implant locations

At the end of each experimental timeline, mice were deeply anesthetized using sodium pentobarbital (10 mg/ml FatalPlus; 100 mg/kg mouse) and transcardially perfused using ice cold 0.1 M phosphate buffer saline (PBS; pH 7.4 with 0.1% heparin) followed by ice cold 4% paraformaldehyde (PFA) in PBS. The entire skull was left in 4% PFA for 48 hr after perfusion. Dissected brains were then cryoprotected at 4° C in a 30% sucrose and 0.1 M PBS solution for at least 48 hr. Brains were then placed in tissue freezing media (Tissue-Tek O.C.T Compound; Sakura Finetek USA), trimmed in 100 μm sections, mounted with nuclear staining (Prolong Antifade Mountant with NucBlue, Life Technologies Corporation), imaged via whole slide scanner (Nanozoomer, Hamamatsu Photonics K.K.). For the current study, only three of eight mice were included for the final analysis due to either loss of headcaps during recordings or inaccuracies in surgery location. Histology of viral expression and optical fiber placement of included mice are found in Supplemental Figure 2.

### 2.4. Fiber photometry

*In vivo* fiber optic recordings were performed using a custom-built fiber photometry system, as previously described (Meng et al., 2018) (Fig. 1e). In the current system, a single laser beam from a 488 nm, continuous wave laser (OBIS 488LS−60, Coherent, Inc.) was aligned into a fluorescence cube and reflected by a dichroic mirror (ZT488/561rpc, Chroma Technology) into a 5-meter-long relay multimode patch cable (M72L05, 200-μm core with 0.39 NA, Thorlabs Inc.) connected via a rotating commutator (optical transmission is >85 ± 3%; RJ1, Thorlabs Inc.) to a 1-meter-long multimode patch cable (M83L01: 200-μm core with 0.39 NA, Thorlabs Inc.) hung from the top of a sound attenuating cubicle (ENV-017M−27, Med Associates Inc.) illuminated by infrared light. Excitation light from the overhead cable was adjusted to ~65 μW before each recording. Working power through the implanted fiber optic cannula was commonly reduced by 75–90% as determined by instances where headcaps came off during recording, exposing the internal fiber optic. Returning emission light in the patch cable then passed through the dichroic of the mirror before entering the spectrometer. Ambient and internally reflected light was controlled by zeroing background noise within the spectrometer’s (QE Pro, Ocean Insight) program (Ocean View, v2.0 +) with the 488 nm laser on and behavioral box closed. Three weeks after surgery (at 11 weeks of age, one week before acquisition records), mice and their home cages were placed in the sound attenuating cubicle once daily for five consecutive days (Fig. 1b). Each day, mice were gently handled using an open palm approximately five to ten times, then briefly scruffed and connected to their implanted fiber optic cannula via a ceramic sleeve (SMCS125S, Precision Fiber Products Inc). After connection, mice were allowed to freely explore their home cage within the behavioral box for five minutes. This habituation procedure was designed to minimize anxiety-induced behavioral artifacts and promote natural wheel running during subsequent experimental recordings. On the fifth day of handling, mice were given a horizontal running wheel (ENV−047, Med Associates Inc.) for the remainder of the study timeline. Four weeks after surgery (at 12 weeks of age, three days after first exposure to the running wheel), mice and their home cages were placed into the sound attenuating cubicle once a day for 60 min for five days. Food, water, and cage enrichment items were removed from the cage to avoid potential catching of the fiber optic cable. A TTL pulse generator (OTPG, Doric Lenses Inc.) was used to frame lock the spectrometer and camera at 25 hz. Mice were recorded again at 15 weeks of age (after one month of daily wheel running) to capture neurotransmission during the maintenance phase.

### 2.5. Behavior annotation

**Fig. 2.**
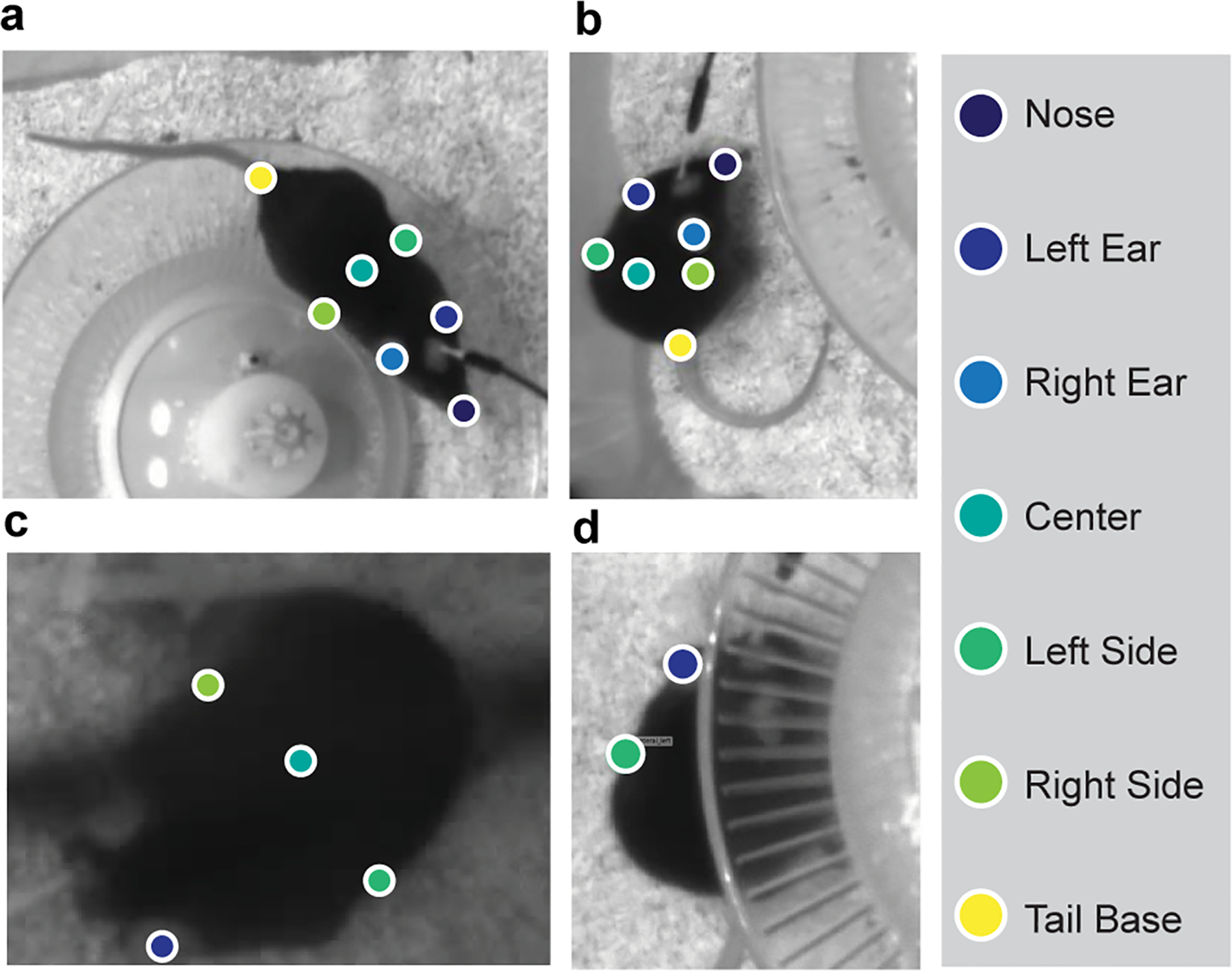
DeepLabCut accurately tracks seven anatomical landmarks during wheel running. a,b) Frames with successful tracking of all seven anatomical landmarks (nose, left/right ear, center, left/right side, tail base). c) Frame with partial occlusions by the fiber optic tether and animal’s body. d) Frame with severe occlusions by the running wheel.

Behavior annotation occurred in three sequential steps: (1) DeepLabCut was used to track the x/y coordinates of body pose estimations (*i*. *e*., anatomical landmarks) for every video frame, (2) these coordinate timeseries were imported into SimBA alongside user-defined ROIs to generate movement and positional features (*e*.*g*., mouse length, width, rotation, proximity to ROIs), and (3) a random forest classifier was trained in SimBA on these newly generated features using example behavioral labels manually annotated in BORIS. To determine a mouse’s location throughout recordings, body pose estimations of the nose, left ear, right ear, left side, right side, center, and tail base were predicted using DeepLabCut v.3 (Mathis et al., 2018). One video from eight mice with non-zero levels of wheel running on the first day of maintenance behavior was used to label anatomical features across 20 frames each (eight videos represented 10% of all data; Extraction Method = Automatic, no user feedback, algorithm = kmeans, and cluster step = 1). Anatomical landmarks were labeled only when clearly visible (Fig. 2a, b). Non-labeled anatomical landmarks were generally due to being under the running wheel, obscuration by the fiber optic tether, or from the mouse itself (Fig. 2c,d). Base settings were used to train the network (Network = resnet_50, Augmentation Method = imgaug, Specific Shuffle Index = 1, User Feedback = No). The network was re-trained by re-labeling incorrect predictions until there were no gross mislabels during all present running events. Common examples of mislabels were flipping of nose/tail base poses and dark bedding spots within the cage. Remaining behavioral videos were then analyzed using this trained model.

**Fig. 3.**
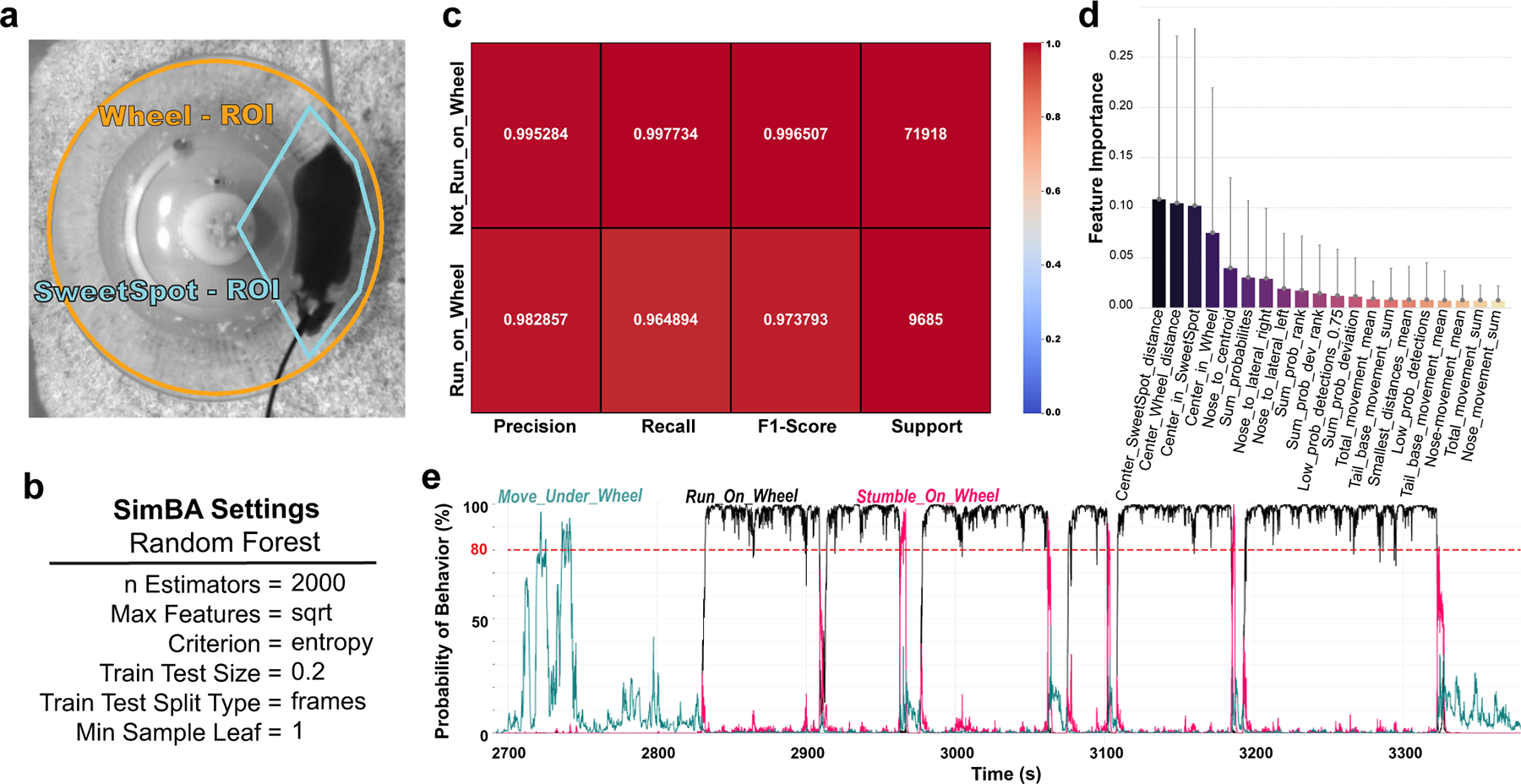
SimBA supervised behavioral classification accurately identifies wheel running behavior. a) Regions of interest (ROIs) manually defined per video: Wheel (outer circumference) and SweetSpot (polygon at the lowest point of the wheel where steady running occurs). b) Random-forest parameters used for training in SimBA. c) Classification-matrix report showing low false-positive rates for running and high true-positive detection, indicating high precision and recall for the running class and near-perfect accuracy for the non-running class. d) Feature-importance profiles showing that spatial proximity to Wheel and SweetSpot ROIs contributed most to Run_On_Wheel classification followed by the length from the nose to left/right sides and body center. e) Example behavior-probability traces for Move_Under_Wheel (cyan), Run_On_Wheel (black), and Stumble_On_Wheel (magenta); peaks in Run_On_Wheel indicate sustained wheel running bouts with high certainty. The horizontal, dashed line represents the probability cutoff to determine the binary designation of each behavior.

The eight videos used to train DeepLabCut were then imported into SimBA v.3.8 (Goodwin et al., 2024) to train a network to label stereotypical wheel running behaviors. When importing, we found best results when no smoothing was applied and pose estimations were excluded if a pose moved 1.5x the average pixel distance from left ear to right ear in subsequent frames. Two regions of interest were set: 1) “Wheel” - the circumference of the wheel and 2) “SweetSpot” - a polygon area around the lowest point of the running wheel where mice would steadily run (Fig. 3a). Desired stereotypical behaviors (Run_On_Wheel, Stumble_On_Wheel, and Move_Under_Wheel) of the eight videos used to train the DeepLabCut network were then manually annotated using BORIS v.9 (Friard and Gamba, 2016). These three behaviors were specifically chosen because they cannot be distinguished by spatial proximity to the Wheel and SweetSpot ROIs alone or by the binary state of wheel rotation. All three behaviors differ only in pose estimation dynamics (*e*.*g*., body length, width, rotation, etc.) in relationship to spatial proximity with the two ROIs. This approach ensures that classification performance reflects unique kinematic features rather than geometric heuristics alone. All behaviors of the training videos were annotated manually via frame-by-frame visual inspection in BORIS (*i*.*e*., given a binary designation of the behavior occurring in that frame or not). Annotation criteria for each behavior were as follows. Because the wheel is oriented horizontally, mice initiate each running event by briefly climbing the ascending slope of the wheel until the wheel accelerates returning their center of mass to the lowest point of the wheel (the “SweetSpot”), where they adopt a quasi-stationary running posture as the wheel rotates beneath them. Run_On_Wheel was annotated from the first frame in which a mouse placed a forepaw on the ascending slope of the wheel followed by consecutive stepping that resulted in translation into the SweetSpot, through the last frame in which the mouse maintained continuous stepping while remaining in contact with the wheel, terminating when the mouse either ceased stepping, left the wheel, or transitioned into another defined behavior. Stumble_On_Wheel was annotated from the first frame in which the mouse’s stepping pattern broke down and the animal was passively displaced out of the SweetSpot while still on the Wheel, until the frame in which coordinated running resumed or the mouse exited the wheel. Move_Under_Wheel was annotated whenever any tracked body part passed beneath the running wheel, with onset and offset defined by the first and last frames in which any body part was located within the Move_Under_Wheel region of interest. Raw manual annotations of these data can be found in our GitHub repository (https://github.com/ThePhysicalActivityMotivationLab/Methods-for-Measuring-Neural-Activity-During-Voluntary-Wheel-Running). The manually labeled videos were partitioned into training (80%) and test (20%) sets by frame, with the test partition held out entirely during model training. Annotated videos from all eight mice, including the five subsequently excluded from photometry analyses, were used to train a random forest classifier using the settings in Fig. 3b. The remaining unlabeled videos were then run through the trained classifier to generate behavior probability traces, with an 80% posterior probability threshold applied as a binary cutoff to retain only high-confidence running events (Fig. 3e).

### 2.6. Data processing and alignment

**Fig. 4.**
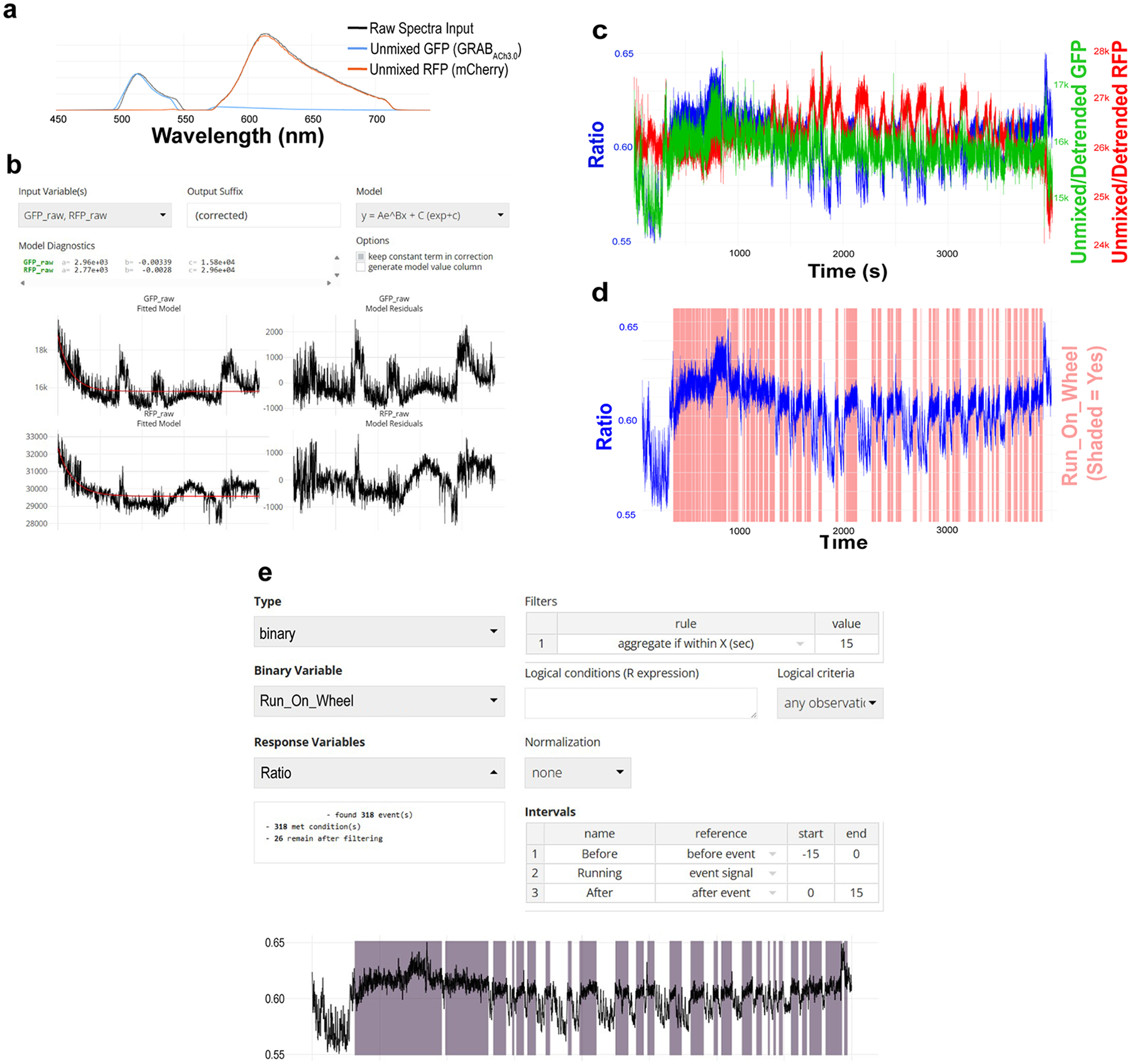
FiPhA processing pipeline integrates spectral signals with behavioral classification for event-based analysis. a) Raw spectrometer signals (black trace) were separated into GRAB_ACh3.0_ (blue trace) and mCherry (red trace) channels using reference spectra acquired from single-color controls. b) Each unmixed channel was corrected for photobleaching/light-switching using an exponential detrending model (exact model parameters shown). c) A GRAB_ACh3.0_:mCherry Ratio was created for every time point to control for artifacts. d) Time-aligned behavior predictions from SimBA (Run_On_Wheel/Not_Run_On_Wheel shown as pink/white shaded regions) were merged with the spectral data. e) Run_On_Wheel was treated as a binary series (purple shaded region). Adjacent bouts separated by ≤ 15 s were merged; bouts lacking ≥ 15 s of non-running both before onset and after offset were dropped.

Data was processed within FiPhA commit 453eca06, an R Shiny framework for analyzing fiber photometry data, similar to Bridge et al., (2024). In short, raw spectrometer data was first unmixed (Fig. 4a) using wavelength traces recorded from mice with only either GRAB_ACh3.0_ (green, an active biosensor used as a proxy for acetylcholine activity) or mCherry (red, a reference fluorophore capturing motion- and blood flow-related signal fluctuations used as a control) expression. Unmixed wavelength values were then “detrended” over time from photobleaching/switching effects using an exponential model (Fig. 4b). A ratio of corrected GRAB_ACh3.0_:mCherry (green:red wavelengths) traces was calculated for all timepoints (Fig. 4c) as a way to control for mechanical bending artifacts of the fiber optic cable and blood flow changes (Zhang et al., 2022).

Wheel running behavior predictions were then merged with raw spectrometer data within FiPhA (Fig. 4d). Wheel running “events” were then binned using a binary Run_On_Wheel determination filtered to reduce behavior noise using the following parameters in order: Run_On_Wheel events within 15 s of each other were merged, and Run_On_Wheel events without 15 s before and after of non-running were dropped if necessary (*e*.*g*., when a mouse was running at the beginning or ending of recording). This length was determined to give ample time between running bouts to avoid confounding baseline values. Absolute ratio values (GRAB_ACh3.0_:mCherry) from 15 s before the initiation of running (“Start”), entire running events (“Running”) and 15 s after the termination of running (“After”) were then exported as RDS files (Fig. 4e).

### 2.7. Preprocessing and descriptive checks

**Fig. 5.**
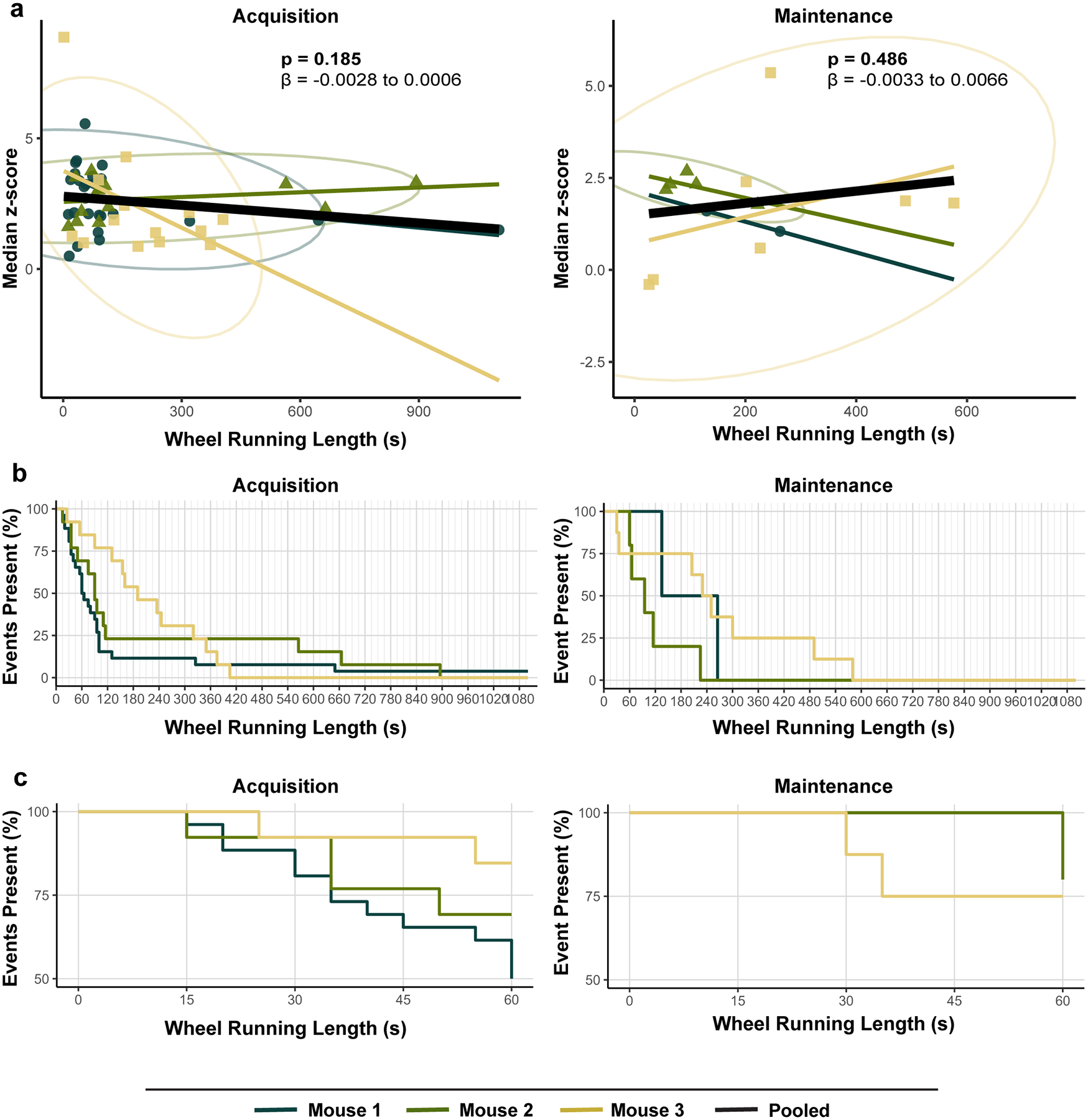
Acetylcholine amplitude in the ventral dentate gyrus is independent of wheel running bout length. a) Relationships between wheel running bout length and median in-running acetylcholine signal (baseline-standardized GRAB_ACh3.0_:mCherry ratio z-score). Black lines show pooled ordinary-least-squares fits across all events (p-values and 95% CI of the pooled slopes are printed). Colored lines show independent within-mouse OLS trends. Ellipses depict 90% data contours per mouse. No reliable association was observed between bout length and acetylcholine amplitude during either phase. b) Wheel running event-length retention curves by mouse during acquisition and maintenance show the percentage of events exceeding a given duration. c) Same retention curves zoomed on 0–60 s to visualize the impact of a 30 s cutpoint: Less than 20% of wheel running events were removed from analysis.

For each wheel running event (*i*.*e*., a single running bout plus the 15 s before initiation and 15 s after termination), the GRAB_ACh3.0_:mCherry Ratio was z-scored across the event window and adjusted to a quiescent baseline (−15 to −10 s before wheel running initiation; +10 to +15 s after wheel running termination). Distributions of wheel running event lengths and median running z-scores for each mouse were inspected for each phase (*i*.*e*., combined 60 min recording sessions across acquisition or maintenance) to confirm comparability (Supplemental Figure S3a,b). To avoid early dropout and tail artifacts when trimming events to uniform lengths for analysis, we first determined if the length of a wheel running event was correlated with the median wheel running event z-score. We fit an ordinary least-squares model with mouse fixed intercepts (median z-score ~ length (s) + mouse) (Fig. 5a). To determine the longest possible wheel running event to trim to while keeping the majority of events, we constructed running event length-retention curves by mouse and phase (Fig. 5b,c). For all downstream analyses, wheel running traces were truncated at 30 s and dropped if shorter as this would retain greater than 80% of total wheel running events.

### 2.8. Acetylcholine during wheel running analyses

For each phase (acquisition and maintenance), all event z-scores from each mouse were averaged into a mouse-level trace (Supplemental Figure 4); these mouse-level traces were then averaged to create a single nested average trace. Statistical comparisons of baseline windows (−15 to −10 s before initiation; +10 to +15 s after termination) to in-wheel running z-scores were performed with paired *t*-tests. To verify baseline window selection, we computed the slope of the acetylcholine signal within consecutive 5-second windows across the peri-event period. The −15 to −10 s pre-initiation window consistently exhibited slopes near zero across all mice and phases, confirming it represents a period of quiescent signal appropriate as a baseline (Supplemental Figure 5). To describe temporal structure *post-hoc*, we binned z-scores of mouse-level traces into consecutive 2.5, 5, and 10 s windows and performed one-way repeated-measures ANOVAs with a Greenhouse-Geisser correction. Pairwise comparisons were reported without correction as adjacent time bins are not independent, and corrections assuming independence would be overly conservative for characterizing a continuous temporal signal. All statistical tests were two-sided with α = 0.05.

### 2.9. Acetylcholine and non-wheel running kinematic analyses

Average acetylcholine activity during each classified event (Run_On_Wheel, Stumble_On_Wheel, and Move_Under_Wheel) was compared to periods without events (No_Event) using session-level mean differences. For each mouse, session-level means were computed separately for each behavior and for No_Event periods across classifier probability thresholds ranging from 0.1 to 0.9, averaged within mouse, and the difference was standardized by the standard deviation of within-mouse differences to compute Cohen’s dz at each threshold. The primary analyses above used the 80% threshold selected *a priori*. Given the proof-of-concept sample size, Cohen’s dz values with 95% CIs are reported as descriptive effect size estimates rather than formal inferential statistics.

To probe z-score fluctuations during off-Wheel events, session-level Spearman’s ρ were calculated for various movement-related kinematics: Center_movement, Mouse_length, Mouse_width, Mouse_area, and Mouse_rotation. Run_On_Wheel_Probability was added and computed across all samples (on and off Wheel) as a positive anchor. Implausible values were hard-trimmed (Center_movement: 0–5; Mouse_area: 50–200; Mouse_length: 15–90; Mouse_width: 15–30; Mouse_rotation: 0–100 on an absolute value scale). Session correlations were Fisher-z transformed and averaged within each mouse, then pooled across mice with equal weight and back-transformed to ρ with 95% CIs.

## 3. Results

### 3.1. Training quality of animal pose estimations and behavior was high

DeepLabCut pose estimation achieved a final training error of 2.46 pixels and a test error of 6.36 pixels, indicating high spatial precision of pose estimation. Visual inspection of pose estimations from videos not included in the training set confirmed quality tracking across wheel and off-wheel contexts. The primary behavior of interest (Run_On_Wheel) achieved high precision (0.983), recall (0.965), and F1-score (0.974) on the held-out test partition (Fig. 3c). Importantly, model performance was strongest for sustained wheel running epochs, where classification probabilities approached ceiling values (Fig. 3e, Supplemental Figure 6c), consistent with the relatively stereotyped kinematics and spatial localization of this behavior. Feature importance analysis indicated that proximity to the “Wheel” and “SweetSpot” ROIs were the dominant predictors (Fig. 3d), followed by pose-derived spatial metrics such as body length. These results are consistent with the spatially constrained nature of wheel running behavior. A follow up test training without ROIs yielded similar precision (0.986), marginally lower recall (0.944), and similar F1-score (0.965) compared to the ROI-inclusive model, indicating that ROIs improve classification sensitivity, but are not strictly required. While feature importance reflects contribution to model splits within the Random Forest framework, it does not imply causal contribution. Visualization of mean SHAP scores can be found in Supplemental Figure 6a. Stumble_On_Wheel recall was lower (0.771), consistent with its relative rarity and greater kinematic variability compared to sustained running (Supplemental Figure 6b).

### 3.2. Acetylcholine in the ventral dentate gyrus rises during wheel running acquisition and maintenance

**Fig. 6.**
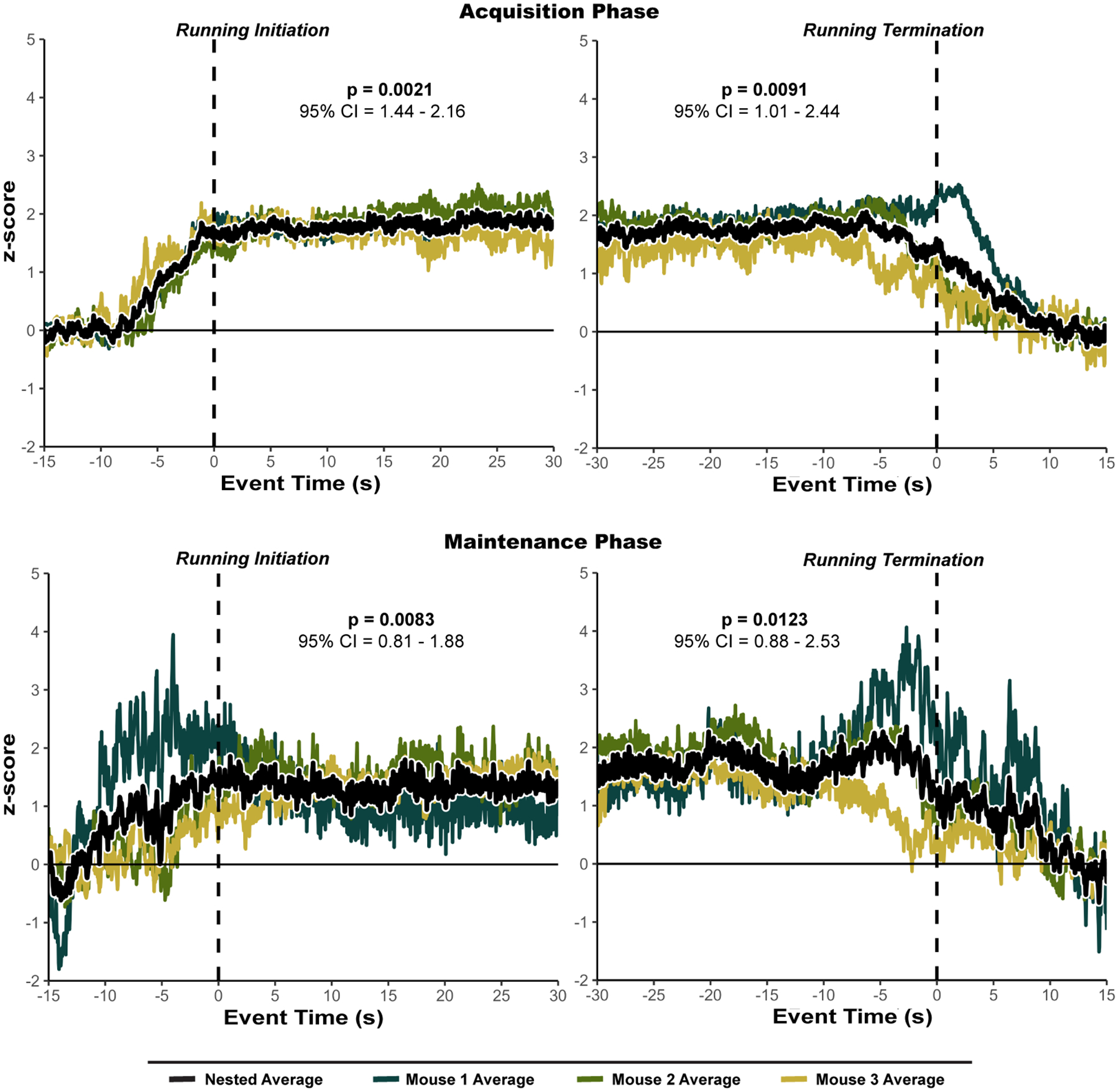
Acetylcholine activity in the ventral dentate gyrus is elevated throughout wheel running. Baseline-standardized GRAB_ACh3.0_:mCherry ratio z-scores show ventral dentate gyrus acetylcholine dynamics aligned to wheel running initiation and termination events, faceted by phase (acquisition and maintenance). Colored traces represent individual mouse event averages. Black traces represent nested averages. Vertical dashed lines mark wheel running initiation or termination. Statistics reflect paired *t*-tests comparing baseline windows (−15 to −10 s before initiation or +10 to +15 s after termination) to in-wheel running acetylcholine z-scores.

**Fig. 7.**
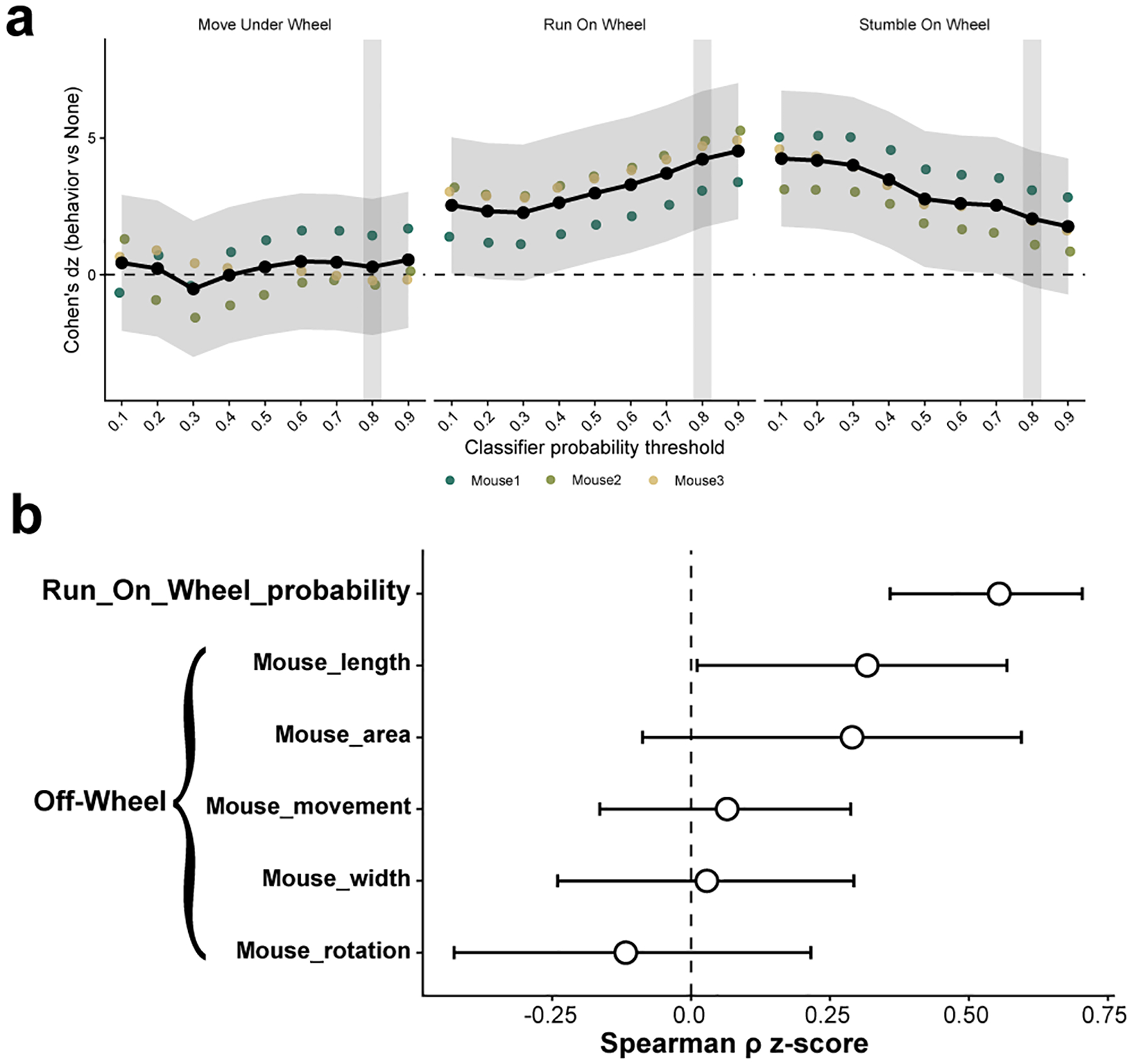
Acetylcholine activity in the ventral dentate gyrus differs across behavioral states by probability threshold and correlates with off-wheel mouse length. a) Cohen’s dz effect sizes for each classified behavioral state versus periods with no labeled event across varying classifier probability thresholds. For each mouse, session-level means were computed separately for each behavior and for None periods, averaged within mouse, and standardized by the standard deviation of within-mouse differences. Points represent the pooled mean Cohen’s dz across mice. Horizontal bars represent 95% CIs. Horizontal dashed line represents no difference (dz = 0). b) Pooled Spearman ρ between GRAB_ACh3.0_:mCherry Z-scores and movement-related features from SimBA. Off-wheel kinematics (bracket) were computed only when On_wheel variable = 0 and were hard-trimmed to exclude impossible values. Points are Fisher-z pooled across mice (equal weight) with 95% CIs. Vertical dashed line represents ρ = 0.

Analyses presented below should be interpreted as a proof-of-concept only as final inclusion sample size was low (N = 3). Statistical tests are presented to characterize effect magnitude, direction, and as an example of how to analyze potential data. Acetylcholine levels increased significantly above baseline during acquisition and maintenance wheel running (Video 1, Fig. 6). During acquisition wheel running, acetylcholine increased relative to baseline at running initiation (p = 0.0021, 95% CI = 1.44–2.16) and remained elevated until termination (p = 0.0091, 95% CI = 1.01–2.44). Similar effects were observed during maintenance wheel running (initiation: p = 0.0083, 95% CI = 0.81–1.88; termination: p = 0.0123; 95% CI = 0.88–2.53). Based on the *post-hoc* time series analyses (2.5, 5, and 10 s bins), acetylcholine activity rose approximately 0–5 s prior to wheel running initiation and remained elevated throughout the bout, and declining within 10–15 s following termination (*post-hoc* comparisons are presented in Supplemental Figure 7). Run_On_Wheel effect sizes increased at higher probability thresholds, suggesting that stricter onset anchoring captures a stronger acetylcholine signal possibly by excluding transitional running frames (Fig. 7a). This phenomenon may explain a portion of the pre-wheel running initiation rise in acetylcholine rather than strictly anticipatory signaling.

Greater effect-size increases during running than during stumbling or moving under the wheel support a physiological rather than artifactual signal (Fig. 7a). Stumble_On_Wheel effect sizes decreased at higher thresholds, suggesting the acetylcholine signal associated with stumbling is weaker and less reliable than that observed during sustained running. Move_Under_Wheel remained near zero across all thresholds. During off-wheel periods, acetylcholine showed positive pooled correlations with mouse length (Fig. 7b, Supplemental Figure 8). No significant associations were found for mouse area, movement, width, or rotation.

## 4. Discussion

We developed a workflow to quantify sub-second neurotransmitter dynamics during voluntary wheel running by aligning fiber-photometry signals with pose-derived behavior. Aligning over 40 h of video with millions of photometry samples, the workflow allowed us to identify running-linked acetylcholine events with an estimated 90% less time than manual annotation while achieving greater than 96% precision and recall. As a proof-of-principle, we observed evidence that acetylcholine in the ventral dentate gyrus rises during wheel running events across both the acquisition and maintenance phases. Off-wheel body length was also positively correlated with acetylcholine activity, though associations were weaker than those observed during wheel running. These findings align with existing literature linking hippocampal cholinergic tone and movement where acetylcholine logarithmically scales with locomotor speed on fast timescales while also reflecting behavioral state and task engagement (Kopsick et al., 2022; Moghadam et al., 2025; Xuan et al., 2025b). Thus, our event-aligned cholinergic transients likely reflect a complex state rather than a purely discrete on/off response to wheel running worthy of further investigation.

A notable outcome is that acetylcholine dynamics were similar across acquisition and maintenance phases. This pattern is consistent with, though not sufficient to conclude due to low n, that acetylcholine activity in the ventral dentate gyrus is not related to motor learning *per se*, but instead reflects neural phenomenon related to physical activity state such as, but not limited to, contextual salience, arousal, or spatial processing demands. Prior studies indicate that hippocampal acetylcholine participates in state-dependent modulation of network dynamics, including interactions with sharp-wave ripples and memory-related processing, in a temporally specific manner (Zhang et al., 2021, 2024). Our data cannot yet separate these possibilities, but they provide a foundation for targeted experiments. Future work should test the functional role of these signals directly as this signal may contribute to physical activity induced plasticity. Opto/chemogenetic modulation of basal forebrain inputs during running or local pharmaceutical blockade would determine whether phasic acetylcholine during voluntary wheel running is necessary for initiating movement, sustaining bouts, reinforcing activity, or impacting physical activity-related memory plasticity. Of note, optogenetic studies manipulating medial septal cholinergic neurons have already shown phase-specific effects on hippocampal activity and non-voluntary wheel running behavior, underscoring the feasibility of this approach (Cassity et al., 2024; Zhang et al., 2021).

Previous findings have found positive correlation between locomotion speed and hippocampal activity. While our data does not include simultaneous wheel running velocity to model speed-signal relationships, future studies would benefit from doing so to compare to off-wheel velocities demonstrated in previous literature (Moghadam et al., 2025; Xuan et al., 2025b). It should be noted that sustained wheel running events identified by SimBA were typically near maximal ambulatory velocity, potentially reducing within-event variance in speed but not eliminating its contribution. Future work should determine whether wheel running is physiologically unique relative to other forms of locomotion (*e*.*g*., cage locomotion or forced treadmill running) with respect to acetylcholine dynamics in the ventral dentate gyrus. Parallel monitoring of other neuromodulators will also be important for situating acetylcholine within the broader motivational circuitry that supports voluntary physical activity and behavioral state regulation.

Three key analytical parameters were examined to evaluate the robustness of the reported signal dynamics during analysis: classifier probability threshold, baseline window selection, and temporal binning resolution. Run_On_Wheel effect sizes increased at higher probability thresholds (Fig. 7a). This raises the possibility that some portion of the rise in acetylcholine activity just before running initiation may represent transitional running stages (*e*.*g*., running from off the wheel onto the wheel or a slow, fragmented walk to run) rather than anticipatory signaling - a distinction that cannot be fully resolved with classifier-based onset detection alone and warrants investigation in future work of the causality of this pre-event signal. The −15 to −10 s pre-initiation window was selected as baseline following a slope analysis confirming it represents a quiescent period across mice and phases (Supplemental Figure 5). Temporal binning at 2.5, 5, and 10 s yielded consistent conclusions of the trends in signal over time (Supplemental Figure 7). Together, these sensitivity analyses support the robustness of the analytical choices made in this study and provide a template for future implementations of the pipeline.

The machine learning-photometry workflow we developed is critical for capturing neural dynamics previously uncharacterized during voluntary wheel running. However, the workflow is intentionally designed to be retrained on study-specific data rather than deployed as a universal pretrained classifier, as laboratory-specific variation will always require adaptation. To repeat the methods presented in this manuscript with similar levels of precision, the entire pipeline must be performed using a study-specific dataset. In our experience, training the classifier on one manually annotated video from a minimum of five mice is sufficient to capture individual variability and generalize to new animals in the testing set. Framerate, contrast, brightness, color spectrum, camera distance, mouse coat color, attached recording apparatus (*e*.*g*., fiber optic or miniscope), and wheel orientation must be matched to maintain performance in DeepLabCut and SimBA. If none of these factors change between cohorts, DeepLabCut and SimBA should maintain performance of newly added videos that are not in the training set. The current study used 60-minute recording sessions, but three sessions of at least 30 min on separate days are likely sufficient. Researchers are recommended to follow documentation and GitHub repositories for each component of the pipeline for step-by-step guidance: DeepLabCut (https://deeplabcut.github.io/DeepLabCut), BORIS (https://boris.readthedocs.io), SimBA (https://github.com/sgoldenlab/simba), FiPhA (https://mfbridge.github.io/FiPhA), and the GitHub repository containing example annotated training data from BORIS, R analysis code, and raw data accompanying this manuscript (https://github.com/ThePhysicalActivityMotivationLab/Methods-for-Measuring-Neural-Activity-During-Voluntary-Wheel-Running).

Although our target cohort included both sexes, attrition from cap loss/misplacements reduced n, limiting statistical power and generalizability; still, the retained mice provided consistent within-mouse effects over many trials (Supplemental Figure 4) as well as strong coherence with previous literature. Due to low n, the current findings should only be considered proof-of-concept of the methodology. The primary failure mode observed in this study was headcap loss, which can be mitigated by thoroughly cleaning and drying the skull surface prior to dental cement application to ensure adequate adhesion. Additionally, chilling the dental cement mixture in ice before application improves ease of application and strength of the adhesion. Fiber optic misplacement, the second highest cause of subject exclusion, reflects surgical variability inherent to stereotaxic procedures; accuracy improves with operator experience and verification of photometry signal quality during the recording session can serve as a real-time indicator of adequate placement. A third potential failure point is animal stress during fiber optic cannula to cable attachment, which may suppress voluntary wheel running during recording sessions. We recommend habituating animals to fiber optic attachment on multiple days immediately prior to recording to minimize stress-related suppression of running behavior (*e*. *g*., two qualifying events from Mouse 1 during maintenance, Supplemental Figure 4). While our workflow utilized fiber photometry, the behavioral classifications can pair with alternative forms of neurobiological measurement such as voltammetry, local field potentials, miniscope, and microdialysis. The primary concern for any planned study is the likelihood of attached wires/tubes twisting in the event mice are spun in circles on the running wheel. In the current study, we avoided damaging the fiber optic cable by using an optical commutator. Overall, the workflow we present here is scalable and can be adapted as preprocessing tools improve. From a translational perspective, these novel methods provide a new way to examine how neurotransmitters and other biomolecules relate to physical activity motivation, affect, and plasticity.

## Supplementary Material

M1

SF1

SF2

SF3

SF4

SF5

SF6

SF7

SF8

## Data Availability

https://github.com/ThePhysicalActivityMotivationLab/Methods-for-Measuring-Neural-Activity-During-Voluntary-Wheel-Running

## Appendix A. Supporting information

Supplementary data associated with this article can be found in the online version at doi:10.1016/j.jneumeth.2026.110839.
