# Supplementary figures and images for: Methods for measuring neural activity during voluntary wheel running
Source: J Neurosci Methods. Author manuscript; Available in PMC 2026 Jul 27. (PMC13404277; doi:10.1016/j.jneumeth.2026.110839)

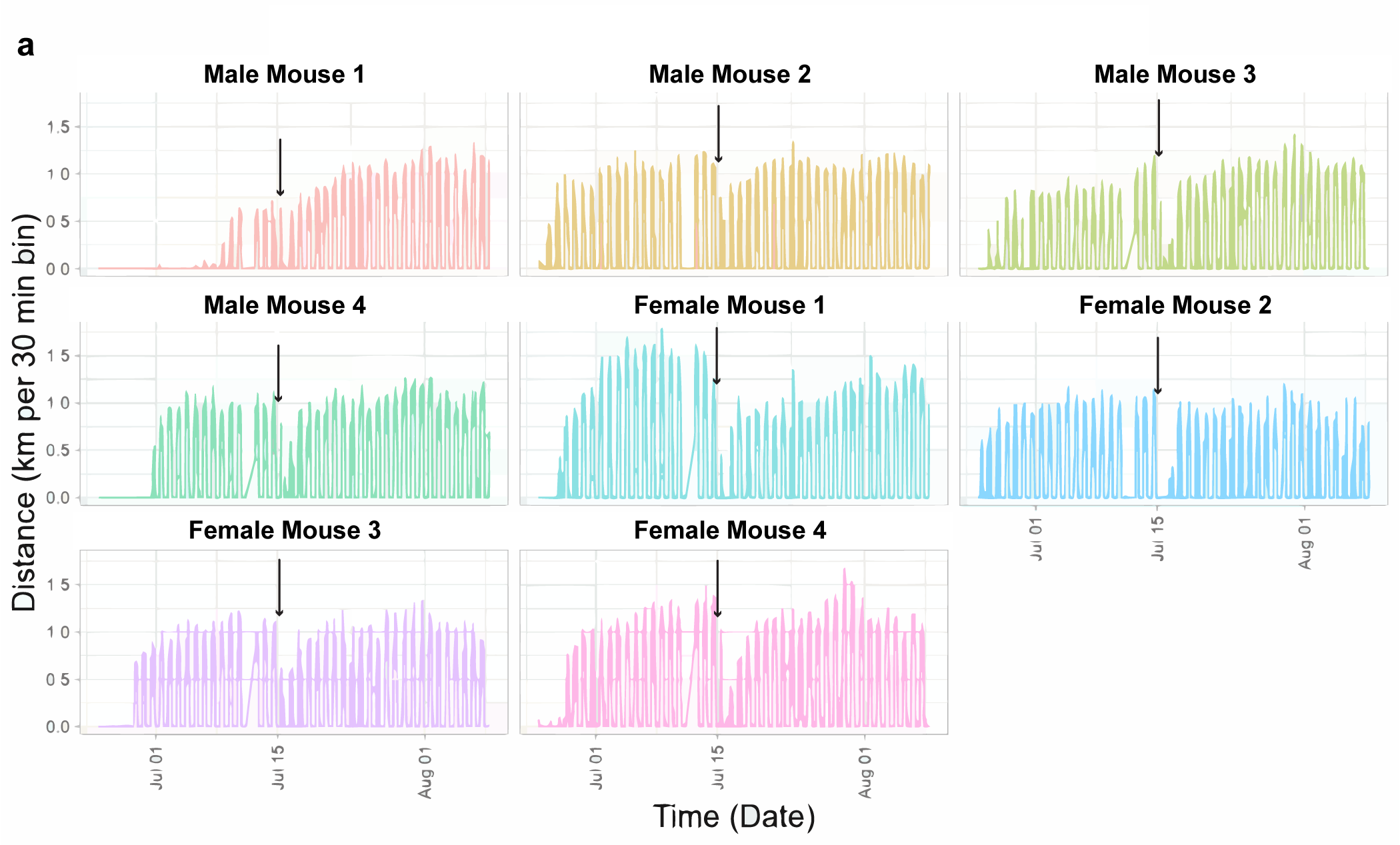

Supplement: SF1 [file NIHMS2194096-supplement-SF1.tif]

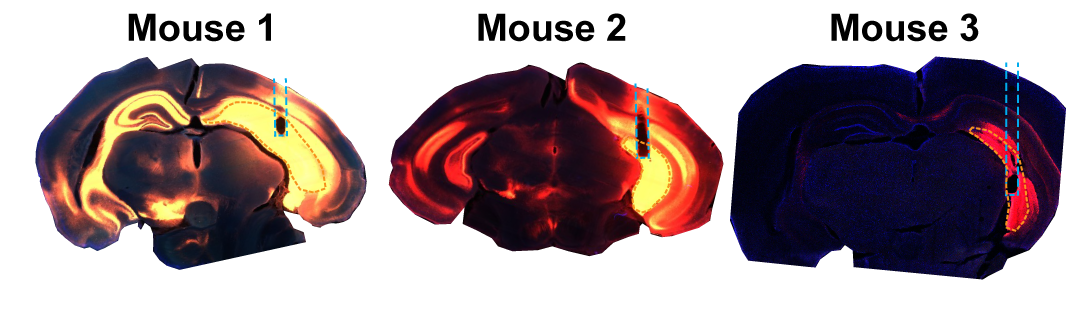

Supplement: SF2 [file NIHMS2194096-supplement-SF2.tif]

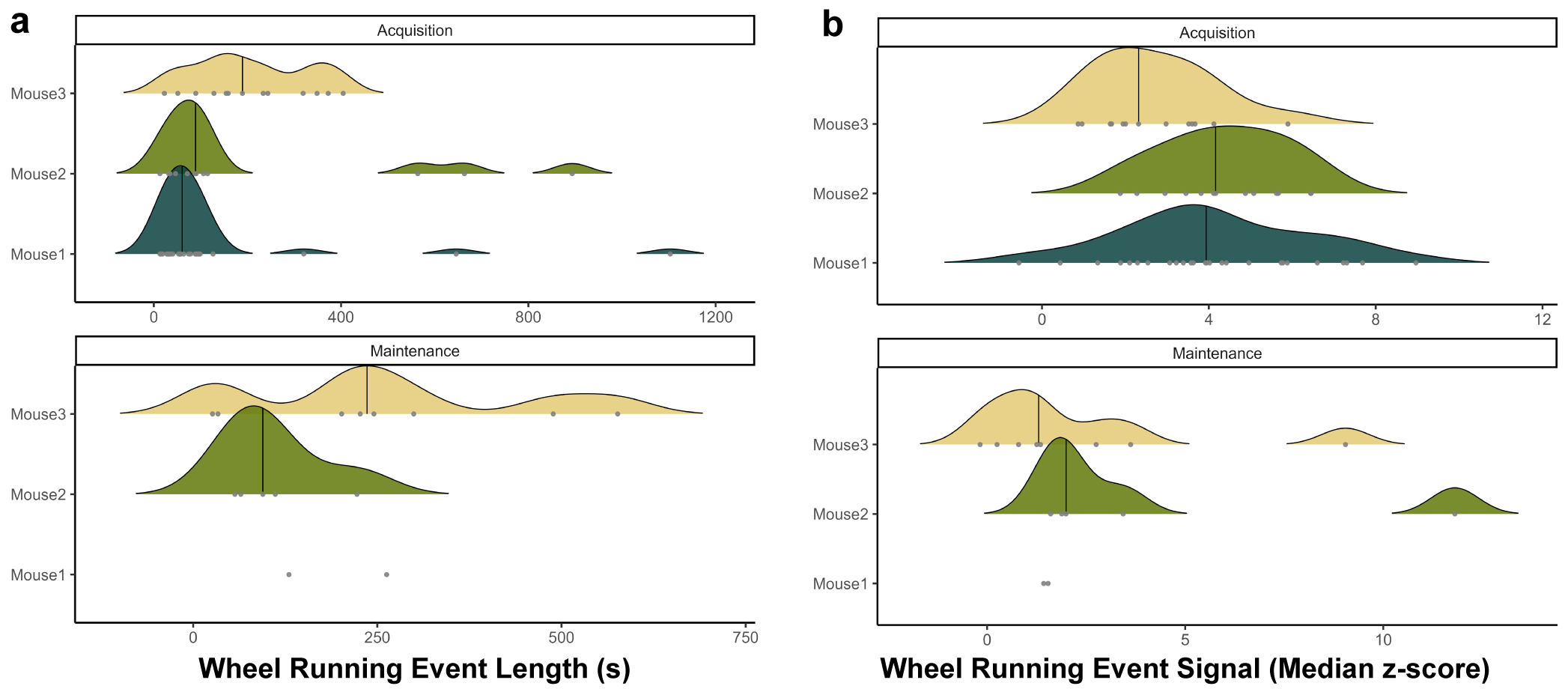

Supplement: SF3 [file NIHMS2194096-supplement-SF3.tif]

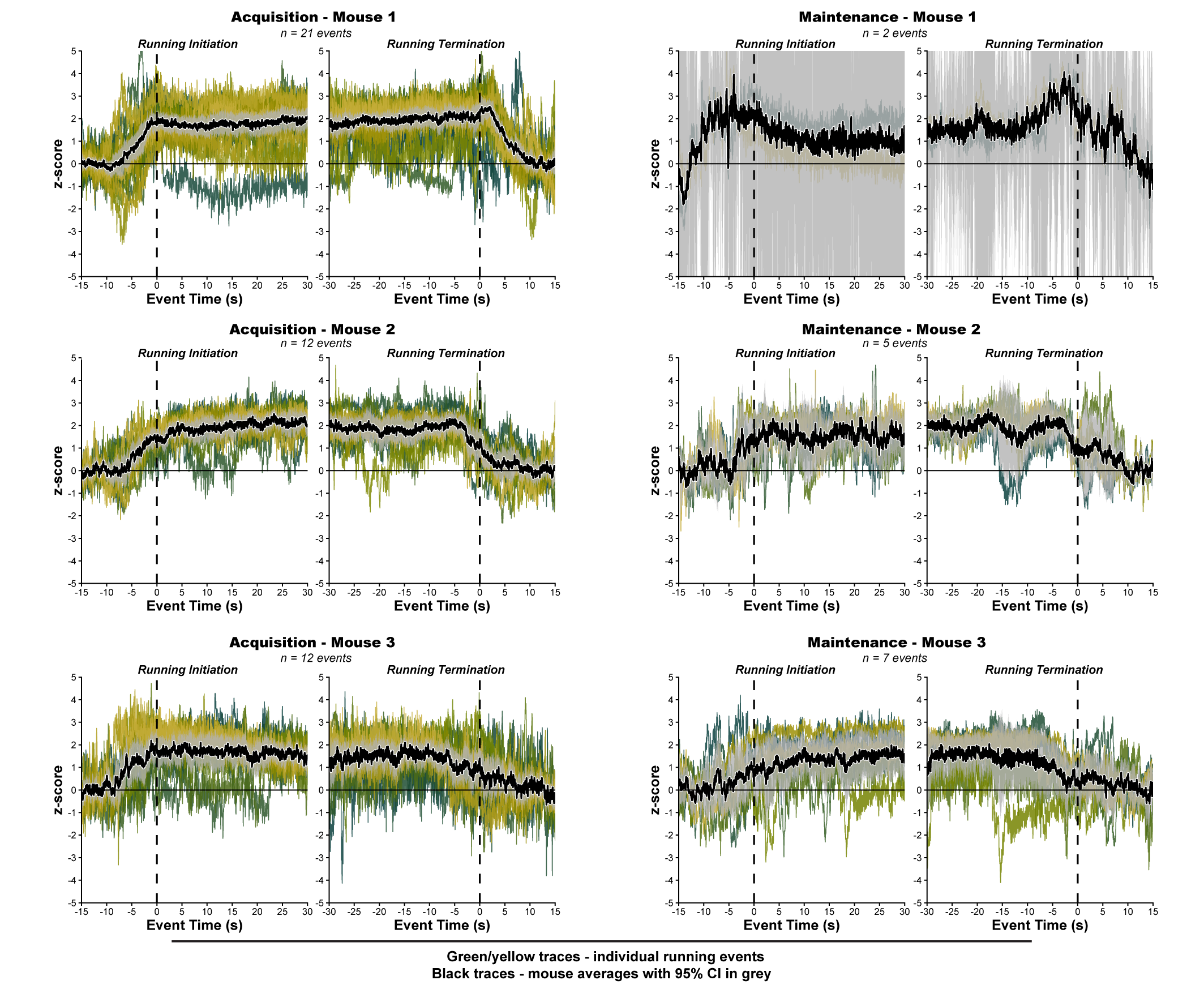

Supplement: SF4 [file NIHMS2194096-supplement-SF4.tif]

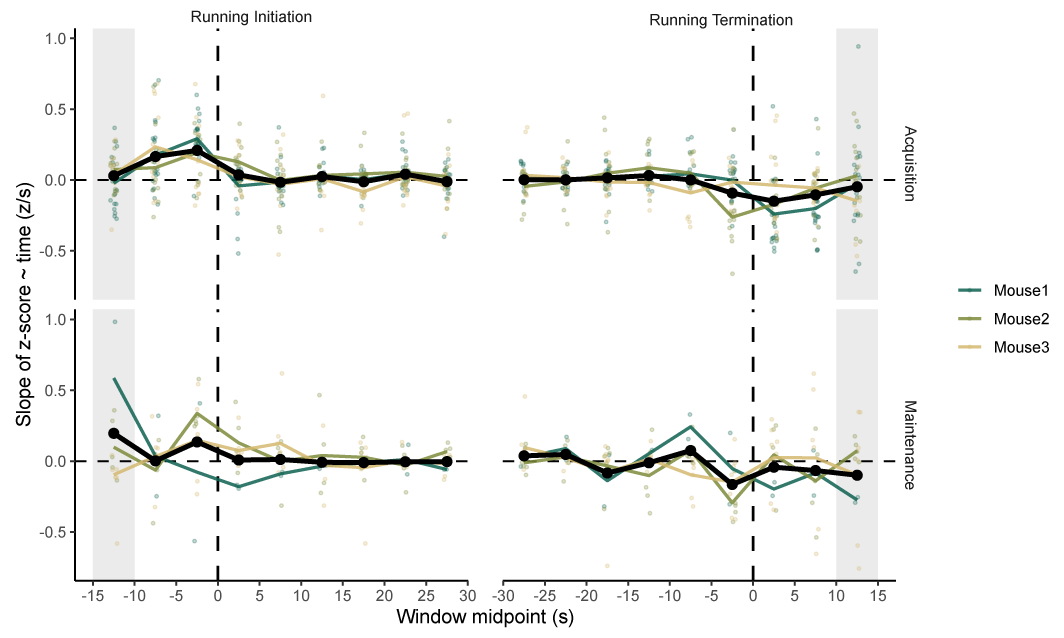

Supplement: SF5 [file NIHMS2194096-supplement-SF5.tif]

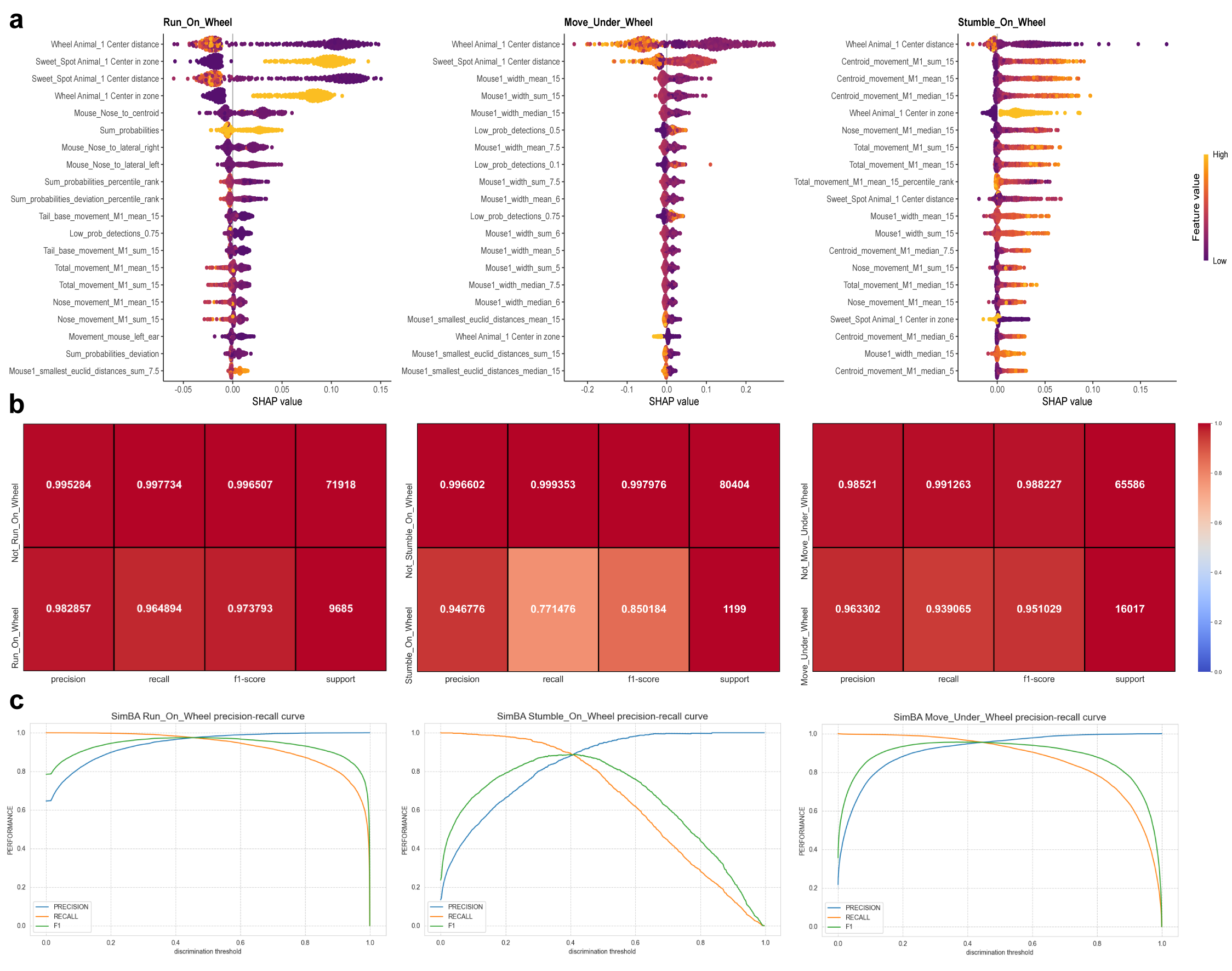

Supplement: SF6 [file NIHMS2194096-supplement-SF6.tif]

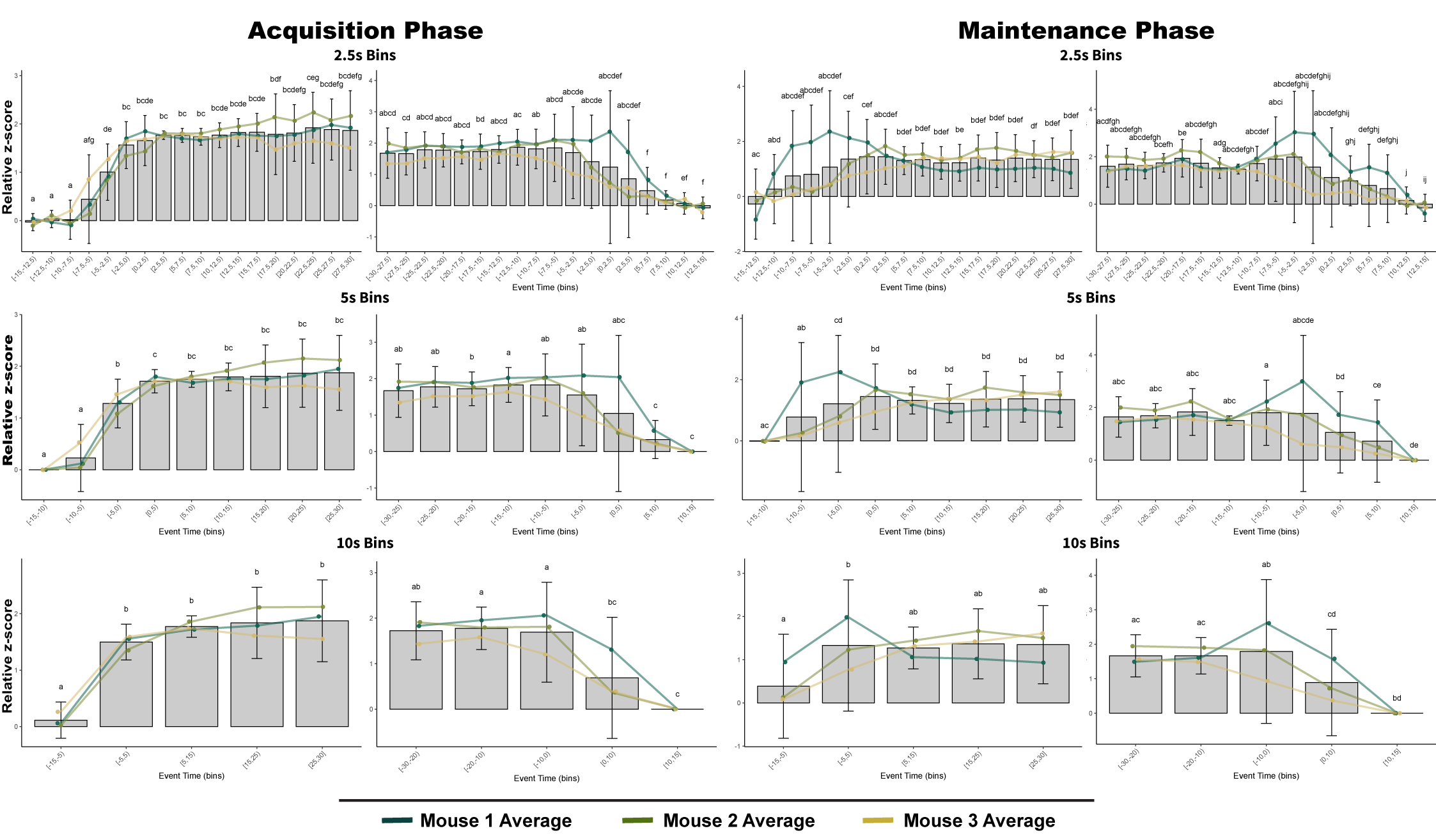

Supplement: SF7 [file NIHMS2194096-supplement-SF7.tif]

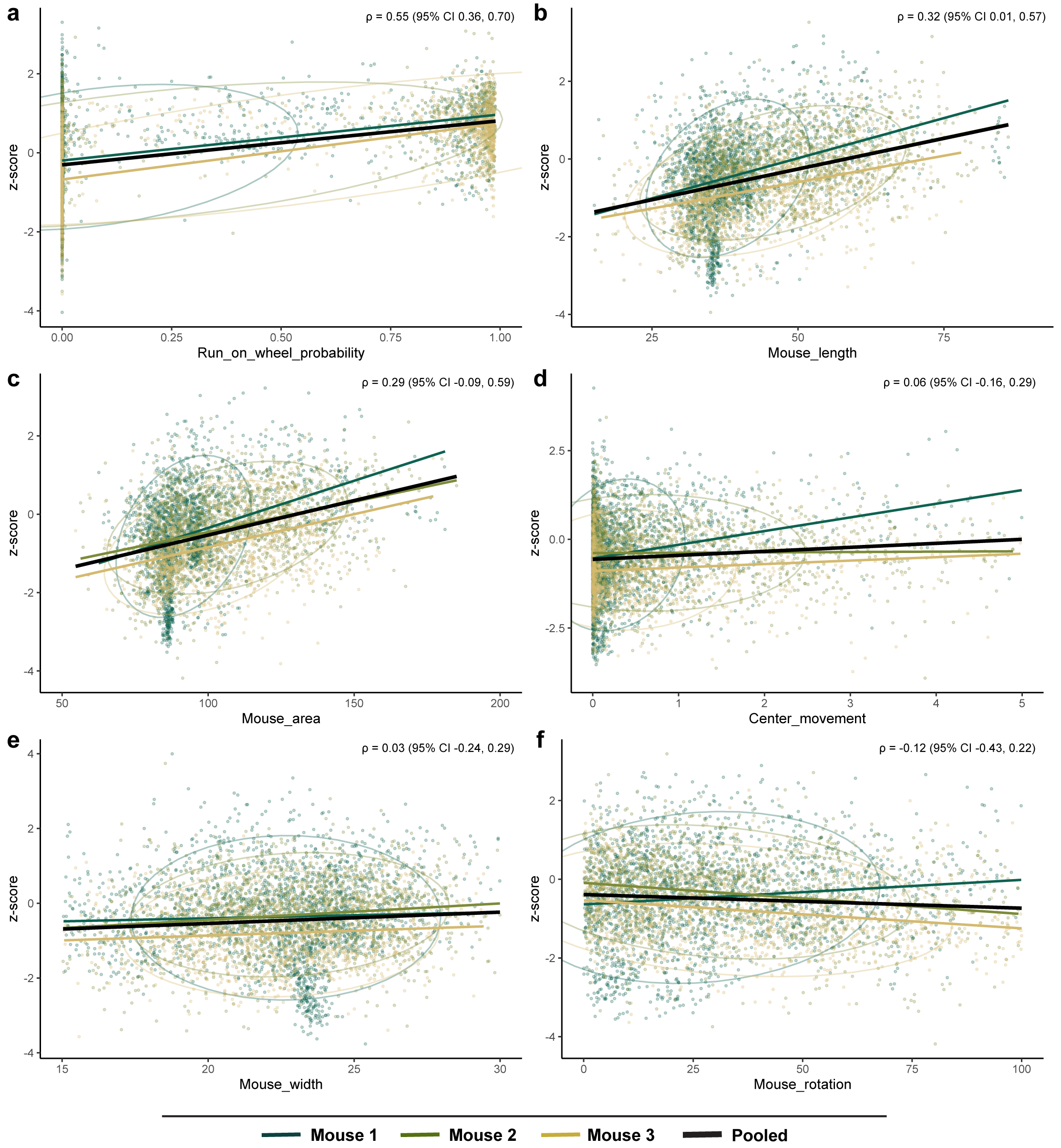

Supplement: SF8 [file NIHMS2194096-supplement-SF8.tif]
